# Influence of Time of Weed Removal on Maize Yield and Yield Components Based on Different Planting Patterns, the Application of Pre-Emergence Herbicides and Weather Conditions

**DOI:** 10.3390/plants14030419

**Published:** 2025-01-31

**Authors:** Dejan Nedeljković, Dragana Božić, Goran Malidža, Miloš Rajković, Stevan Z. Knežević, Sava Vrbničanin

**Affiliations:** 1 University of Belgrade, Faculty of Agriculture, Nemanjina 6, 11080 Belgrade, Serbia; dejanbrus@gmail.com (D.N.), dbozic@agrif.bg.ac.rs (D.B.); 2 Institute of Field and Vegetable Crops, 21000 Novi Sad, Serbia; goran.malidza@ifvcns.ns.ac.rs; 3 Institute of Medicinal Plant Research “Dr. Josif Pančić”, Tadeuša Košćuška 1, 11000 Belgrade, Serbia; rajkovicmilos@gmail.com; 4 Department of Agronomy and Horticulture, University of Nebraska-Lincoln, Lincoln, NE 68728, USA; sknezevic2@unl.edu

**Keywords:** maize, planting pattern, twin-row, CTWR, yield

## Abstract

The crop yield can be affected by many factors, including various levels of weed presence. Therefore, we conducted a study to evaluate the effect of time of weed removal in combination with planting pattern and pre-emergence-applied herbicides on maize yield and yield components in 2015, 2016 and 2017. The experiments were designed in a split–split plot arrangements with three replications, which consisted of the two main plots (standard/conventional and twin-row planting pattern), two subplots (with and without pre-emergence herbicide application) and seven sub-subplots (seven weed removal timings). In the dry season of 2015, maize yield was much lower (413–9045 kg ha^−1^) than in the wet 2016 seasons with yields of 5759–14,067 kg ha^−1^ across both planting patterns. Yield and yield components were inversely correlated with the time of weed removal. The application of pre-emergence herbicides delayed the critical time for weed removal (CTWR), which ranged from V4 to V10 and from V3 to V11 for standard and twin-row planting patterns, respectively. Herbicides also protected various yield components, including 1000 seeds weight and number of seeds per cob.

## 1. Introduction

Maize (*Zea mays* L.) is one of the three most grown and cultivated plants in the world (alongside rice and wheat), and is the leading field crop in Serbia with a production area of about 1 million ha [1]. Maize is grown as a row crop, with an open canopy during early growth stages, which affects crop development, yield quantity and quality [2,3,4].

The effect of weeds is well documented in the literature. For example, season-long competition by *Datura stramonium* at densities of 1, 3, 6, and 10 plants m^−1^ of row decreased maize yield by 11–57% [5], while 1, 2, 4 and 8 plants m^−1^ of *Abutilon theophrasti* decreased maize yield by 1.2–39.3% [6]. According to Soltani et al. (2016) [3], the average potential maize yield loss caused by weeds in the United States is 50%. Teasdale and Cavigelli [7] determined a variability in the degree of yield reduction due to weed competition from 4 to 76% based on a six-year experiment.

As maize yield loss caused by weed competition depends on many factors, the same authors found that the highest yield losses were obtained in years with below-average rainfall, while the lowest losses were obtained in years with above-average rainfall. Based on a database of trials for herbicide efficacy carried out over 27 years with 205 environmental conditions, Landau et al. [8] analyzed 69 potential variables affecting maize yield loss using machine learning techniques. They concluded that late-season control of weeds is one of the most important predictors of maize yield loss. Therefore, to prevent yield losses due to late weed removal, it is necessary to determine the critical time of weed removal (CTWR), which represents a period in the crop growth cycle during which weeds must be controlled [9].

The concept of twin-row planting makes it possible to increase crop density using planters designed to plant two (twin) rows 15–25 cm apart with the mid-points of the two sets of twin rows spaced at conventional row centers of 75–100 cm (30–40 inches) [10], which corresponds to a final maize stand of about 95,000 plants ha^−1^. However, some research and extension articles do not report significant yield differences between the two planting systems (conventional versus twin row) when the plant populations are the same [11,12]. In contrast, some popular press articles and sales brochures have promoted twin-row planting as a way to increase crop yields in comparison to single-row planting [13,14].

Knezevic et al. [9] defined the CTWR as the maximum time period during which crops can tolerate early-season weed competition before yield reduction becomes irrevocable, which has been studied by many researchers around the world. Based on an overall analysis in the USA and their own research, Page et al. [15] stated that the CTWR most often occurs when the maize crop is in the stage of having three to five developed leaves. Several authors [16,17,18,19] suggested that the CTWR could be significantly affected by the application of pre-emergence (PRE) herbicides. For example, Ulusoy et al. [19] reported that the CTWR without pre-emergence herbicides begins at the V3 maize stage compared to the V5 stage with the application of atrazine, while treatment with saflufenacil/dimethenamid-P + pyroxasulfone pushed the CTWR to the V8-V10 stage. Nedeljković et al. [18] also reported the CTWR in standard and twin-row planting pattern ranged from V1 to V2 and V2 to V3 without pre-emergence herbicide treatment, while pre-emergence herbicides (S-metolachlor + terbutylazine) application delayed the CTWR to the V4–V10 and V3–V11 leaf stages of maize, respectively. Also, Barnes et al. [20] reported that the CTWR in popcorn ranged from V4 to V5 in the absence of pre-emergence herbicides, but when atrazine/S-metolachlor was applied as the pre-emergence herbicide, the CTWR was delayed until V10 to V15. The EU Green Deal aims to reduce the use and risks of pesticides by 50% by 2030, which include pre-emergence herbicides on maize. But, it is still unclear whether a reduction in herbicide use could be a real solution in sustainable crop production [21]. Less rainfall due to climate change in the future will affect the efficacy of common pre-emergence herbicides. However, utilizing combinations of pre-emergence herbicides with additional non-chemical measures and compliance with the CTWR will create a more sustainable IWM system and help corn production adapt to more extreme weather [22]. Aspects of weed management and planting techniques have been studied previously, but this research uniquely combines these factors to evaluate their cumulative effects under varying agro-ecological and climatic conditions. Based on the above, the main hypothesis in these studies is that yield and yield components will depend on time of weed removal, planting pattern, pre-emergence herbicides and weather conditions. Therefore, the objective of this study was to evaluate the effect of the time of weed removal in different planting patterns (standard and twin row) with and without pre-emergence herbicides on yield and yield components of maize in Serbia.

## 2. Results

### 2.1. Temperature and Precipitation

Maize yield depends on many factors including weather conditions, especially precipitation. Hatfield et al. [23] indicated that both insufficient and excessive precipitation during the period of the grain-filling have a negative impact on crop yield. Also, the extreme maximum temperatures and diurnal temperature range indices have a negative impact, while the extreme precipitation event indices have positive correlations with the yield of the rainfed crops [24].

In this study, the average month temperatures used were the 30-yr average across the three seasons, but precipitation was significantly different between years (Table 1). The results show that 2015 was the most unfavorable year for maize production, while 2017 is classified as an average year, and 2016 was one of the most favorable years for maize production in the agro-ecological conditions of Serbia. Since rainfall differed between the years, results for yields and yield components were presented separately for each year.

### 2.2. Maize Yield

**Figure 1:**
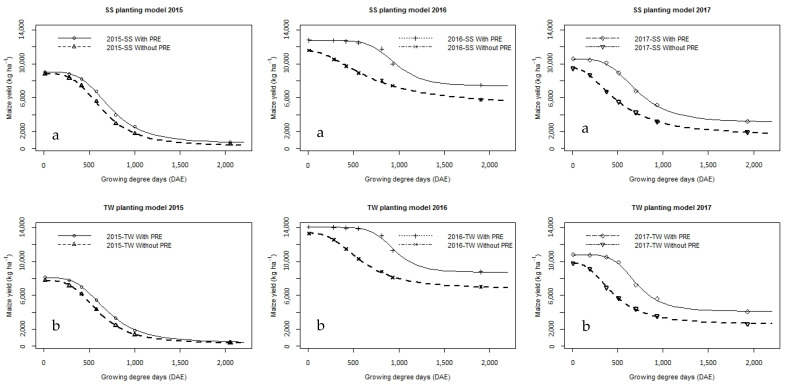
Maize yield response to increasing duration of weed interference as represented by growing degree days (days after emergence, DAE) grown (**a**) in standard (SS) and (**b**) twin-row (TW) planting patterns without and with pre-emergence herbicides, in Padina, Serbia.

Yield decreased exponentially with increasing duration of weediness in all seasons (Figure 1, Table 2). In general, the highest yields were found in the weed-free control (PRE/TW: 8133, 14,067 and 10,792 kg ha^−1^; PRE/SS: 9045, 12,803 and 10,594 kg ha^−1^; without PRE/TW: 7752, 13,308 and 9783 kg ha^−1^; and without PRE/SS: 8807, 11,588 and 9466 kg ha^−1^), and the lowest were found in the plots with season-long weeds (PRE/TW: 550, 8788 and 4075 kg ha^−1^; PRE/SS: 777, 7490 and 3197 kg ha^−1^; without PRE/TW: 413, 6999 and 2610 kg ha^−1^; and without PRE/SS: 517, 5758 and 1921 kg ha^−1^) in the first, second, and third season, respectively. However, the differences in yield between the planting patterns were less prominent, with the yield in TW being higher in the second and third seasons, which was the reverse of the first season.

In all seasons (first, second and third), the grain yield was higher in the variant with PRE-applied herbicides (TW: 550–8133 kg ha^−1^; SS: 777–9045 kg ha^−1^; TW: 8788–14,067 kg ha^−1^; SS: 7491–12,803 kg ha^−1^; and TW: 4076–10,792 kg ha^−1^; SS: 3198–10,594 kg ha^−1^, respectively) than in the variant without PRE-applied herbicides (TW: 413–7753 kg ha^−1^; SS: 517–8807 kg ha^−1^; TW: 6999–13,308 kg ha^−1^; SS: 5759–11,588 kg ha^−1^; and TW: 2611–9784 kg ha^−1^, SS: 1922–9466 kg ha^−1^, respectively) in both planting patterns.

Despite the differences in maize yields due to the three tested factors (explained above), there were also differences in maize yields due to different levels of precipitation between seasons. This is in accordance with the findings of Dragičević et al. [25] who indicate that higher precipitation results in a higher yield of maize grain in the agro-ecological conditions of Serbia. In the weed-free control plots, the grain yield was the lowest in the first season (PRE/TW: 8133 kg ha^−1^; PRE/SS: 9045 kg ha^−1^; without PRE/TW: 7752 kg ha^−1^; and without PRE/SS: 8807 kg ha^−1^), followed by the second (PRE/TW: 14,067 kg ha^−1^; PRE/SS: 12,803 kg ha^−1^; without PRE/TW: 13,308 kg ha^−1^; and without PRE/SS: 11,588 kg ha^−1^) and third (PRE/TW: 10,792 kg ha^−1^; PRE/SS: 10,593 kg ha^−1^; without PRE/TW: 9783 kg ha^−1^; and without PRE/SS: 9466 kg ha^−1^) seasons, in both maize planting patterns and pre-applied herbicide options. The same trend is recorded in treatments with different times of weed removal (Figure 1).

### 2.3. Yield Components

**Figure 2:**
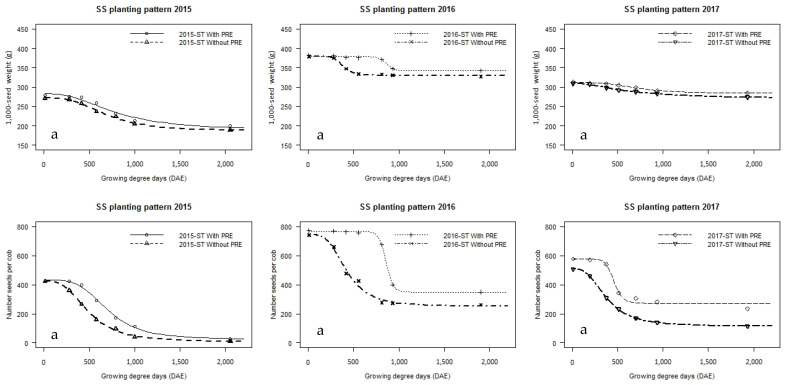

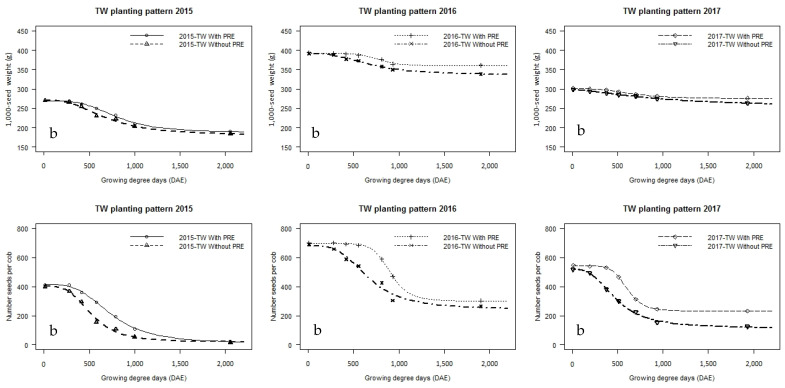
Maize yield components’ ((**a**) 1000 seed weight and number of seeds per cob in SS; (**b**) 1000 seed weight and number of seeds per cob in TW) responses to increasing duration of weed interference as represented by growing degree days (d after emergence, DAE) grown without and with pre-emergence herbicides in standard (SS) and twin-row (TW) planting patterns, in Padina, Serbia.

Seed number per cob decreased exponentially with increasing duration of weediness, especially in plots without pre-emergence herbicides (Figure 2a,b; Table 3). The lowest number of seeds per cob occurred in the first season (2015). Without pre-emergence herbicides, the number per cob was the lowest in both planting regimes (SS: 11–423; TW: 13–396) compared to much higher values in plots with pre-emergence herbicides (SS: 25–425; TW: 22–412). When compared between the two planting regimes, in the absence of pre-emergence herbicides, the number of seeds in the cob was higher in TW than in SS by 1.6–25.0% depending on the time of weed removal, while in the presence of pre-emergence herbicides, this parameter was higher in SS than in TW by 3.3 to 4.3%.

The number of seeds per cob was significantly higher in the second season (2016) than in the first season across both planting patterns and regardless of whether pre-emergence herbicides were used or not. Without pre-emergence herbicides, the number of seeds per cob ranged from 660 to 274 in SS and from 659 to 305 in TW, with no significant differences (0.2%) between the planting patterns used in the treatment with the earliest times of weed removal. However, as the time of weed removal became later, the difference between SS and TW became more pronounced, and at the latest time of weed removal, the number of seeds per cob was 10.1% higher in TW than in SS. On the contrary, the number of seeds per cob was higher in plots with pre-emergence herbicides, ranging from 773 to 348 in SS to 702 to 301 in TW. In the third season, variations in the number of seeds per cob were the same as those in the second season (Table 3).

The 1000 seed weight (Figure 2a,b; Table 3) was influenced by planting pattern, pre-emergence herbicides, time of weed removal and season. In general, seed weight decreased as the duration of weediness increased. Across all seasons, the application of pre-emergence herbicides helped to increase the 1000 seeds weight compared to the variant without pre-emergence herbicides. Although this parameter was the highest in the second and the lowest in the first season, pre-emergence herbicides prevented a reduction in the 1000 seed weight. In all seasons, the values for plots with pre-emergence herbicides (first, SS: 200–278 g; TW: 191–270 g; second, SS: 343–383 g; TW: 361–394 g; third, SS: 285–313 g; TW: 276–301 g) were slightly higher in comparison with the values in plots without pre-emergence herbicides (first, SS: 189–271 g; TW: 189–270 g; second SS: 327–379 g; TW: 339–391 g; third, SS: 273–309 g; TW: 263–298 g). In the first season, there were no significant differences in the values of this parameter between SS and TW planting pattern, and in second season, the 1000 seed weight was significantly higher in TW than in SS, while in the season with the average rainfall (third) this parameter had higher values in SS than in TW.

## 3. Discussion

Out of all the factors tested in this study, time of weed removal had the highest effects on maize yields, followed by presence of pre-emergence herbicides, while the planting pattern had the least impact.

The significant impact of time of weed removal on maize yield has been affirmed by several authors. Tursun et al. [26] confirmed this result for three maize types (field maize, popcorn, sweet maize) in Turkey. Also, Adamič Zamljen and Leskovsek [27] showed that increasing the period of weed interference significantly reduced maize yields (22 to 41%) in different tillage regimes in Slovenia. The positive effect of pre-emergence herbicides on maize yield could be attributed to a delay in CTWR. For example, the application of pre-emergence herbicides on popcorn resulted in yield increase, because the herbicides were effective in controlling most weeds, and thus reduced weed interference [19]. A similar effect of pre-emergence herbicides on yield of glyphosate-resistant maize was confirmed by Ulusoy et al. [19]. Planting maize in double rows increases the grain yield per hectare thanks to the higher number of ears per plant and the percentage weight of the second ear grains compared to the yield [28].

Our findings about the maize yield’s response to time of weed removal are like those of others, who confirmed that the duration of weed interference affected crop development and yield [25,29]. Although the loss of yield depended on the time of weed removal, yield had the same trend in SS and TW, and a more pronounced decrease was recorded in SS, which confirms that in optimal seasons, growing maize in TW provides a higher grain yield [30]. This is similar to the results of Gözübenli [31], who states that grain yield and planting pattern are correlated with agro-ecological conditions and maize genotype. This supports previous claims that optimal soil moisture has a positive effect on crop yield, especially at higher seeding rates [12]. Satterwhite et al. [32] also agree with this statement, pointing out that, in the TW pattern, available moisture can be a key factor for increasing yield. Contrary to this, in arid areas or dry years, to achieve a high yield in the TW pattern, it is necessary to provide irrigation. The occurrence of drought in the grain-filling phase of maize development is very often reflected in a decline in the yield [33,34]. Due to the water deficit (Table 1) that occurred in the critical phases of crop development (reproductive phase), grain yield was the lowest in the first season.

The application of pre-emergence herbicides significantly delayed CTWR in both planting patterns, with delays ranging from two to nine leaf stages across the years (Table 2). For example, in the SS planting pattern, applications of pre-emergence herbicides delayed CTWR from the V2 to the V4 stage, the V1 to the V10 stage and the V1 to V5 stages, for the first, second and third seasons, respectively (Table 2). A similar trend occurred in the TW pattern. However, the differences in CTWR between the planting patterns were not as evident for each herbicide factor (Table 2). Therefore, the overall differences in the occurrence of CTWR can be mainly attributed to the differences in the presence or absence of pre-emergence herbicides, and to a lesser degree to planting pattern, which is consistent with the results of other researchers [18,19,35,36]. Barnes et al. [20] also concluded that pre-emergence atrazine/S-metolachlor delayed the CTWR in popcorn for up to eight leaf stages (32 days). Also, the residual activity of atrazine and saflufenacil/dimethenamid-P + pyroxasulfon delayed CTWR in glyphosate-resistant maize for up to eight fewer stages (21 day) [18]. The efficacy of pre-emergence herbicides is affected by many factors, including weather and soil conditions. Temperature and soil moisture directly affect the behavior of herbicides by influencing herbicide solubility, movement and degradation. Our study showed the impact of different weather conditions on the practical implementation of the CTWR concept, while their incorporation into different pedo-climatic conditions should be evaluated in future studies [37]. PRE application herbicides are a valuable tool in any weed control arsenal. Their ability to prevent weeds before they emerge, long-term effectiveness and environmental benefits make them suitable for sustainable agricultural production and healthy environments. In modern sustainable agriculture the use of systemic and pre-emergence herbicides are increased [38]. Yield components that are most sensitive to weed duration and presence of herbicide treatments should demonstrate primary control over observed differences in crop yield. The treatments did not affect maize density or cob number per plant. However, there were significant effects on seed number per cob and seed weight.

Differences in seed weight between seasons are also likely attributed to differences in rainfall precipitation. Others also reported that lack of water affected yield components (including 100-seeds weight) [33]. Although, the 1000 seed weight in TW was similar (first season) or lower (third season) than in SS, the total seed weight in TW was greater as the number of maize plants per unit area in TW was higher (around 15%) than in SS, which justifies such a crop planting pattern [28,39].

## 4. Materials and Methods

Field experiments were conducted in 2015, 2016 and 2017 in Padina (South Banat, Serbia), on silty clay loam soil (pH 7.18 to 8.2; humus 2.74–3.85%). Before planting, fields were conventionally tilled, including plowing at a depth of 25–30 cm with the addition of NPK (15:15:15) fertilizer (300 kg ha^−1^), followed by pre-sowing soil preparation with the addition of 200 kg ha^−1^ of fertilizer KAN (27% of N). Maize was seeded in the period between the middle and end of April depending on the experimental year. Seeding rates were 80,000 (ST) and 93,900 (TW) seed ha^−1^. There was no irrigation applied. Weather data (Table 1) were obtained from the nearest weather station at a distance about of 10 km from the experimental field. The total rainfall during the 1st, 2nd and 3rd growing seasons at the experimental site was 281.1, 526.4 and 336.0 mm, respectively. In relation to the long-term average of precipitation (399.6 mm), the rainiest season was the 2nd growing season (about 30% more rain than the long-term average), compared to the moderately rainy 3rd season (about 19% less rain than the long-term average), while the 1st season was the driest (about 30% less rain). In addition, the distribution of precipitation during the 2nd season was significantly more favorable compared to the other two seasons (Table 1). In all three growing seasons, average air temperatures were similar to the long-term average (20 °C).

Major weed species present it the experiments included grassy species (*Sorghum halepense*) and broadleaves (*Helianthus annuus*, *Cirsium arvense*, *Datura stramonium*, *Convolvulus arvensis*, *Amaranthus retroflexus*, *Chenopodium album*, *Solanum nigrum*, *Brassica napus*, *Xanthium strumarium*) at the densities of 35–143 plants m^−2^ (for more details on species composition see [18]). Experiments were set up in a split–split plot design with three replications, which consisted of two main plots, two subplots, and seven sub-subplots. The two main plots were (1) the single-row planting pattern (ST; with a spacing of 70 cm × 25 cm) and (2) the twin-row planting pattern (TW; with spacing of 20 cm × 25 cm and between twins of 50 cm). The two subplots included PRE herbicide application, (1) without PRE herbicide and (2) with PRE herbicide, and were separated from each other by a 1 m distance. PRE herbicide (S-metolachlor (1.44 kg a.i. ha^−1^) + terbuthylazine (0.75 kg a.i. ha^−1^)) was applied between seeding and emerging time, using an Amazone UF 1201 sprayer equipped with TeeJet XR 11,003 flat-fan nozzles (Spraying System Co., Wheaton, IL, USA), calibrated to deliver 300 L ha^−1^ solutions at 200 kPa. Sub-subplots consisted of seven weed removal timings where weeds were allowed to grow until predetermined growth stage of maize: (1) season-long weedy plots, (2) three leaves (V3), (3) six leaves (V6), (4) nine leaves (V9), (5) fifteen leaves (V15), (6) beginning of flowering (VT) and (7) season-long weed-free growth. The average maize development stage was determined by counting fully developed leaves from 10 consecutive plants in each plot. The weeds were removed as needed by hand hoeing. Individual plot sizes were 10 m × 4.2 m (ST) and 10 m × 4.4 m (TW), with six crop rows or twin rows in each experimental unit. Six meters from the mid part of two central rows of maize were used to measure yield and yield components, meaning that 2 m were left out on both sides to avoid plot edge effects.

The regression analysis was used to relate the time of weed removal (*x*-axis) to the actual crop yield (*y*-axis). The number of growing degree days (GDDs) accumulated from crop emergence was used as the time unit to quantify the duration of weed presence and length of the weed-free period (*x*-axis). As the rate of crop development is well correlated with the thermal time, the GDDs factor is a more biologically meaningful measure of the amount of time needed for plant growth and development than some other indicators (e.g., days or weeks after crop emergence); thus, it was utilized for practical purposes for determining the CTWR. Furthermore, GDDs can also be useful for comparing data from different planting dates, locations and years, as it provides a continuous and precise scale for the *x*-axis [9]. As the reference point for the accumulation of GDDs, the day after maize emergence (DAE) was used. Temperatures were recorded hourly throughout the growing season and GDDs were calculated using the equation described by Gilmore and Rogers [40]:

GDD = Σ [(Tmax + Tmin)/2 − Tbase] (1)

where Tmax and Tmin are the daily maximum and minimum air temperatures (°C), and Tbase is the base temperature (10 °C) for maize growth.

The final maize harvest was conducted soon after physiological maturity. Samples for yield determination were obtained by hand-harvesting the middle two rows (third and fourth rows), each 6 m long in every plot, and the cobs were shelled and adjusted to 14% moisture. Yield components (1000 seeds weight, number of seeds per cob and cob length) were also determined.

Data analyses were performed in the R program [41] utilizing the “drc” statistical add-on package [42]. A four-parameter log-logistic regression model was used to calculate yield and yield components [42]:

Y = C + (D − C)/{1 + exp [B (log X − log E)]} (2)

where Y is the response (e.g., yield), C and D are lower and upper limit, B is the slope of the curve at the inflection point, X is GDDs calculated after maize emergence and E is the GDDs, giving a 50% response between the upper and lower limit (also known as the inflection point, ED_50_).

The difference between the parameters for each combination of planting pattern, PRE herbicides and season (weather conditions) was also determined by comparing the standard errors (±SE) and *t*- and F-tests at the 5% significance level.

## 5. Conclusions

The time of weed removal, in combination with pre-emergence herbicides, planting pattern (SS and TW) and weather conditions, affected maize yield and yield components (1000 seed weight and number of seeds per cob). In the dry season (first) in both planting patterns, the yield was much lower than in seasons with average precipitation (second and third). Maize yield components were inversely correlated with the time of weed removal in all three seasons and variants with and without pre-emergence herbicides. In the dry (first) season, CTWR without pre-emergence herbicides occurred at the V2 stage, while the application of pre-emergence herbicides delayed CTWR to the V4 and V3 stages in the SS and TW planting patterns, respectively. During the wet season (second one), CTWR without pre-emergence herbicides occurred at the V1 and V2 stages compared to the V10 and V11 stages in the pre-emergence herbicide plants, for SS and TW, respectively. These results reaffirm the well-known benefits of pre-emergence herbicides for not just controlling early emerging weeds, which are the most competitive against the crop, but also for protecting yield and yield components.

## Figures and Tables

**Table 1:** Average air temperature and total precipitation during 2015, 2016 and 2017 growing seasons and the 30-yr average in Padina, Serbia.

	Average Air Temperature				Total Precipitation			
Month	2015	2016	2017	30-yr Average	2015	2016	2017	30-yr Average
	°C				mm			
April	13.5	15.5	12.7	13.6	5.1	76.1	3.0	51.5
May	19.1	17.5	18.4	18.2	63.9	88.7	125.9	72.3
June	21.9	22.5	24.3	21.9	43.5	117.3	94.1	95.6
July	26.8	24.4	25.9	23.8	7.0	123.5	43.0	66.5
August	26.0	22.3	26.1	23.8	82.5	87.6	32.1	55.1
September	20.0	19.7	18.4	18.5	79.1	33.2	37.9	58.6
Season	21.2	20.3	21.0	20.0	281.1	526.4	336.0	399.6

**Table 2:** Regression parameters (±SE) and CTWR for 5% reduction in maize yield in two planting pattern models and variant with/without pre-emergence herbicides in 1st–3rd season in Padina, Serbia.

Season	Application of Herbicides	Planting Pattern	Regression Parameters (SE)				Parameters of CPWR			
							GDD	DAE	Growth Stage	
			B	C	D	I_50_				
1st	With PRE	SS	3.9 (0.2)	629.4 (105.5)	9048.9 (70.3)	728.7 (10.1)	342.5 (4.8)	26	BBCH 14	V4
	Without PRE	SS	3.5 (0.2)	279.4 (126.6)	8822.2 (97.9)	647.4 (12.4)	279.1 (5.4)	21	BBCH 12	V2
2nd	With PRE	SS	6.3 (1.6)	7384.7 (261.8)	12,759.6 (130.4)	957.3 (27.9)	599.9 (17.5)	58	BBCH 20	V10
	Without PRE	SS	2.0 (0.1)	5060.9 (214.8)	11,479.2 (123.0)	702.1 (40.3)	161.1 (9.3)	16	BBCH 11	V1
3rd	With PRE	SS	2.8 (9.8)	3033.5 (120.9)	10,603.1 (73.7)	713.3 (12.7)	321.8 (5.7)	36	BBCH 15	V5
	Without PRE	SS	2.1 (0.2)	1422.6 (85.0)	9496.4 (518.9)	518.9 (10.3)	127.7 (2.5)	16	BBCH 11	V1
1st	With PRE	TW	−3.6 (0.1)	0.6 (0.7)	95.3 (0.9)	688.6 (6.8)	306.1 (8.1)	22	BBCH 13	V3
	Without PRE	TW	−3.4 (0.1)	0.4 (0.7)	96.4 (0.9)	606.1 (6.5)	255.8 (7.1)	18	BBCH 12	V2
2nd	With PRE	TW	−7.1 (0.6)	0.2 (0.8)	38.0 (1.5)	949.5 (20.4)	627.1 (13.8)	61	BBCH 21	V11
	Without PRE	TW	−2.7 (0.1)	−0.04 (0.7)	49.4 (1.0)	592.9 (13.9)	202.6 (11.2)	20	BBCH 12	V2
3rd	With PRE	TW	−5.0 (0.7)	−0.1 (1.4)	61.8 (2.4)	703.8 (22.2)	392.8 (30.1)	41	BBCH 16	V6
	Without PRE	TW	2.5 (0.1)	0.7 (1.0)	74.2 (1.4)	473.9 (11.9)	147.6 (9.8)	19	BBCH 12	V2

SS (standard planting pattern); TW (twin-row planting pattern). Regression parameters (±SE) showing the slope (B), lower limit (C), upper limit (D) and 50% reduction (I_50_). GDD (growing degree days); DAE (days after emergence).

**Table 3:** Regression parameters (±SE) showing the slope (B), lower limit (C), upper limit (D), and growing degree days (GDDs) at 50% reduction (I_50_) in different maize yield components for no and with pre-emergence herbicides in two planting patterns in 1st–3rd season in Padina, Serbia.

Season	Application of Herbicides	Planting Pattern	Yield Components	Regression Parameters (SE)			
				B	C	D	I_50_
1st	With PRE	SS	1000 seed weight	2.5 (0.4)	187.9 (8.5)	283.1 (5.0)	786.8 (94.2)
			Seeds per cob	3.9 (0.4)	23.1 (14.3)	433.0 (9.4)	697.1 (23.7)
	Without PRE	SS	1000 seed weight	3.0 (0.5)	186.0 (4.8)	271.8 (3.1)	689.2 (42.3)
			Seeds per cob	3.0 (0.2)	4.6 (8.9)	424.3 (7.9)	490.2 (13.9)
2nd	With PRE	SS	1000 seed weight	23.7 (2.7)	342.9 (3.3)	378.8 (1.7)	859.9 (22.9)
			Seeds per cob	23.3 (1.3)	348.1 (5.9)	766.5 (2.9)	857.6 (3.5)
	Without PRE	SS	1000 seed weight	7.1 (1.7)	329.9 (1.4)	379.2 (2.3)	385.4 (13.9)
			Seeds per cob	3.5 (0.7)	250.3 (25.4)	745.2 (27.9)	425.2 (30.4)
3rd	With PRE	SS	1000 seed weight	3.6 (0.8)	284.1 (1.9)	311.8 (1.1)	710.4 (46.0)
			Seeds per cob	9.8 (3.9)	271.6 (17.5)	576.4 (19.8)	458.1 (23.2)
	Without PRE	SS	1000 seed weight	1.8 (0.3)	269.2 (3.9)	309.9 (1.4)	625.2 (84.7)
			Seeds per cob	2.9 (0.1)	112.4 (2.5)	508.8 (2.5)	377.5 (3.7)
1st	With PRE	TW	1000 seed weight	3.7 (0.4)	187.7 (3.0)	269.6 (1.9)	798.3 (27.1)
			Seeds per cob	3.4 (0.3)	8.9 (10.6)	415.6 (6.9)	736.2 (19.2)
	Without PRE	TW	1000 seed weight	2.7 (0.5)	179.4 (7.5)	271.2 (4.3)	693.0 (65.7)
			Seeds per cob	3.6 (0.6)	17.4 (19.2)	400.9 (16.2)	524.0 (30.5)
2nd	With PRE	TW	1000 seed weight	7.2 (12.5)	360.1 (2.9)	391.7 (4.6)	780.0 (158.6)
			Seeds per cob	8.8 (0.8)	300.2 (6.4)	697.1 (3.3)	903.9 (6.6)
	Without PRE	TW	1000 seed weight	2.8 (0.5)	335.9 (3.8)	391.4 (2.3)	679.0 (51.1)
			Seeds per cob	3.4 (1.1)	243.5 (43.5)	681.2 (33.1)	667.5 (70.8)
3rd	With PRE	TW	1000 seed weight	3.2 (0.3)	275.1 (0.9)	300.9 (0.5)	658.2 (22.8)
			Seeds per cob	6.8 (0.3)	231.3 (2.9)	543.6 (2.2)	601.6 (4.6)
	Without PRE	TW	1000 seed weight	1.5 (0.2)	250.6 (4.8)	297.9 (0.7)	982.7 (146.4)
			Seeds per cob	2.9 (0.3)	112.9 (11.5)	517.9 (8.9)	483.6 (17.6)

SS (standard planting pattern); TW (twin-row planting pattern). Regression parameters (±SE) showing the slope (B), lower limit (C), upper limit (D), and 50% reduction (I_50_).

## Data Availability

Data are contained within the article.
